# Statistical Analysis of High-Dimensional Genetic Data in Complex Traits

**DOI:** 10.1155/2015/564273

**Published:** 2015-08-04

**Authors:** Taesung Park, Kristel Van Steen, Xiang-Yang Lou, Momiao Xiong

**Affiliations:** ^1^Department of Statistics, Seoul National University, Gwanak_1 Gwanak-ro, Gwanak-gu, Seoul 151-747, Republic of Korea; ^2^Montefiore Institute, Université de Liège, Bâtiment B28, Office 0.15-B37, Grande Traverse 10, 4000 Liège, Belgium; ^3^Department of Biostatistics, University of Alabama at Birmingham, Ryals Public Health Building, No. 420B, Birmingham, AL 35294, USA; ^4^Division of Biostatistics, Human Genetics Center, School of Public Health, The University of Texas Health Science Center at Houston, 1200 Herman Pressler, Houston, TX 77030, USA

With the recent development of high-throughput DNA microarray and next-generation sequencing techniques for detecting various genomic variants (SNVs, CNVs, INDELs, etc.), genome-wide association studies (GWASs) have become a popular strategy to discover genetic factors affecting common complex diseases. Many GWASs have successfully identified genetic risk factors associated with common diseases and have achieved substantial success in unveiling genomic regions responsible for the various aspects of phenotypes.

However, identifying the underlying mechanism of disease susceptible loci has proven to be difficult due to the complex genetic architecture of common diseases. The previously associated variants through GWASs only explain a small portion of the genetic factors in complex diseases. This rather limited finding is partly ascribed to the lack of intensive analysis on undiscovered genetic determinants such as rare variants and gene-gene interactions. Unfortunately, standard methods used to test for association with single common genetic variants are underpowered for detection of rare variants and genetic interactions.

This special issue is dedicated to presenting state-of-the-art statistical and computational methods for finding missing heritability underlying complex traits with massive genetic data including GWAS, next-generation sequencing, and DNA microarray data. The main focus of this special issue is on data mining and machine learning for advanced GWAS analysis. The advanced GWAS analysis includes multi-SNP analysis, gene-gene and gene-environment interaction analysis, estimation of missing heritability, and analysis of population heterogeneity. This special issue provides a platform to the researchers with expertise in data mining to discuss recent advancements in analytic approach of post-GWAS association analysis in field of statistics and bioinformatics.

The paper by W. Lee et al. proposes an approach to identifying clinically interesting subgroups in a heterogeneous population. The identification step uses a clustering algorithm and proposes an improved false discovery rate- (FDR-) based measure to remedy the overestimation of the ordinary FDR-based approach. The paper by Y. Kim et al. performs heritability estimation by using population- and family-based samples. The main idea lies in utilizing genetic relationship matrix to parameterize the variance of a polygenic effect for population-based samples.

Three other papers consider gene-gene and gene-environment analysis. First, J. Yee et al. proposed interaction analysis for quantitative traits using entropy. Although there have been several methods proposed for gene-gene interaction using entropy, this is a robust entropy-based gene-gene interaction analysis that does not necessarily require an assumption on the distribution of trait for quantitative traits. Second, S. Y. Lee et al. focused on identifying multi-SNP effects or gene-gene interactions for survival phenotypes. In the framework of the multifactor dimensionality reduction (MDR) method, several extensions for the survival phenotype are considered and compared to the earlier MDR method through comprehensive simulation studies. Third, the paper by H. Xu et al. proposes a new GWAS strategy for detecting gene-gene and gene-environment analysis by combining the generalized multifactor dimensionality reduction-graphics processing unit (GMDR-GPU) algorithm with mixed linear model approach. It was further employed to investigate the genetic architecture of important quality traits in rice. The reliability and efficiency of the model and analytical methods were verified through Monte Carlo simulations.

The next two papers discuss multi-SNP analysis. Y. J. Yoo et al. propose a new multi-bin linear combination (MLC) test for multiple SNP analysis. It first performs clustering analysis to find cliques, complete subnetworks of SNPs with all pairwise correlations above a threshold, and then performs MLC test. Through simulation studies, the clique-based algorithm was shown to produce smaller clusters with stronger positive correlation than other MLC tests. The paper by S. Won et al. focuses on comparing penalized and nonpenalized methods for disease prediction with large-scale genetic data. It was shown that penalized regressions are usually robust and provide better accuracy than nonpenalized methods for disease prediction.

Next, the work of J. Joo et al. considers robust genetic association tests for GWAS. How these robust tests can be applied to the replication study of GWAS and how the overall statistical significance can be evaluated using the combined test formed by *p* values of the discovery and replication studies were demonstrated.

Finally, the paper by L. Li and M. Xiong proposes a dynamic model for RNA-seq data analysis. To extract biologically useful transcription process from the RNA-seq data, the ordinary differential equation (ODE) model was proposed for modeling the RNA-seq data. Differential principal analysis was developed for estimation of location-varying coefficients of the ODE.

This special issue discusses the most challenging issues in multiple SNPs approaches including gene-gene interaction and introduces statistical and computational methods for data mining and machine learning for revealing hidden association network of genotype-phenotype relationship. The nine papers in this special issue provide scientists with an overview on the recent advancements in multiple SNP analysis for GWASs. We hope the papers can encourage researchers towards a more extensive use of statistical genetics and bioinformatics techniques for research in biology and medical sciences.

